# Photoinduced Electron Transfer Based Ion Sensing within an Optical Fiber

**DOI:** 10.3390/s111009560

**Published:** 2011-10-11

**Authors:** Florian V. Englich, Tze Cheung Foo, Andrew C. Richardson, Heike Ebendorff-Heidepriem, Christopher J. Sumby, Tanya M. Monro

**Affiliations:** Institute for Photonics & Advanced Sensing (IPAS) and School of Chemistry & Physics, The University of Adelaide, North Terrace, SA 5005, Australia; E-Mails: tze.foo@adelaide.edu.au (T.C.F.); andrew.richardson@adelaide.edu.au (A.C.R.); heike.ebendorff@adelaide.edu.au (H.E.-H.); christopher.sumby@adelaide.edu.au (C.J.S.); tanya.monro@adelaide.edu.au (T.M.M.)

**Keywords:** fluorescence sensor, fiber-optic sensor, microstructured optical fiber (MOF), photo induced electron transfer (PET), metal ion

## Abstract

We combine suspended-core microstructured optical fibers with the photoinduced electron transfer (PET) effect to demonstrate a new type of fluorescent optical fiber-dip sensing platform for small volume ion detection. A sensor design based on a simple model PET-fluoroionophore system and small core microstructured optical fiber capable of detecting sodium ions is demonstrated. The performance of the dip sensor operating in a high sodium concentration regime (925 ppm Na^+^) and for lower sodium concentration environments (18.4 ppm Na^+^) is explored and future approaches to improving the sensor’s signal stability, sensitivity and selectivity are discussed.

## 1. Introduction

Small-core microstructured optical fibers (MOFs) with relatively large air holes surrounding the core can serve as a convenient platform to enable close interaction of liquids, loaded into the holes, with the portion of the guided light located in these voids [1]. Such liquids can contain optically and/or chemically active materials like organic fluorophores or quantum dots that facilitate interrogation of the species of interest, at nL–μL volume scales [2].

Lead-silicate (F2, Schott glass, n = 1.62) suspended-core MOFs can be manufactured [3] using low-temperature billet-extrusion techniques and active pressurization for inflation of the fiber holes, as described in the supplementary material. The use of inflated fibers results in shorter filling times than uninflated fibers. This fiber-based platform provides access to long optical interaction lengths, and typically 10–30 cm fiber lengths are used. A significant fraction of the guided mode field is located within the holes of a suspended-core fiber, enabling strong interaction between the guided light and the species of interest, which is loaded into the holes [1,4]. Thus, suspended-core soft-glass MOF enable the development of small, flexible and cost-effective sensing architectures and their suspended nanowire design provides a much more rugged sensing platform compared to “free-standing” nanowires [5]. Such MOFs open up possibilities for improved performance and the development of new, compact fiber optic sensors based on absorption or fluorescence spectroscopy techniques.

Recently, there has been considerable interest in the development of fiber optic ion-sensors for chemical, medical, biological and environmental applications [6–9]. The first use of microstructured fibers for fluorescence-based metal-ion detection (Al^3+^) was reported by Warren-Smith *et al.* [10]. Fluorescence detection based ion sensors have the potential to offer a simple, sensitive, selective and fast measurement alternative to traditional ion-selective electrodes [11,12]. Potential applications for MOF-based ion sensors lie in the area of small-volume ion sensing in confined spaces or precision point measurements, e.g., ion-sensing in the root-zone of plants for monitoring and optimization of agricultural processes [13]. Also, such ion sensors could be used for distributive sensing applications [14] such as geo-chemical mapping or the highly sensitive on-site assessment of water quality [15].

The PET effect is well-established tool for fluorescent molecular sensing [16–18]. PET-type fluoroionophores provide an intramolecular electron-transfer mechanism that results in signaling systems with natural fluorescence switching ability [16]. The mechanism of PET-based fluorescence ion sensors can be described in terms of the molecular orbital (MO) energies and redox potentials of the ionophore and fluorophore [19,20]. During cation sensing, the cation is coordinated within the ionophore and this coordination inhibits the quenching effect of the ionophore, resulting in an increase in the quantum yield. Successful commercial PET sensors include: sodium [21], potassium [22] and calcium [23] sensing.

In this article we report the first demonstration of a PET-based microstructured optical fiber dip-sensor. The sensor principle was tested for the small-volume detection of sodium ions. Furthermore, the sensitivity and signal stability of these sensors were investigated, including a study of the photobleaching response and photostability of the PET-fluoroionophore system.

## 2. Experimental

### 2.1. Synthesis of the Model PET-Fluoroionophore

**Scheme 1.**
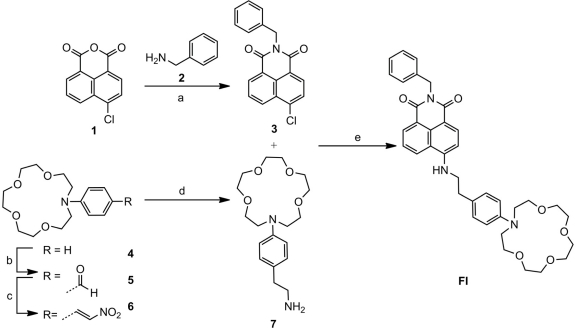
The synthesis of the PET-fluoroionophore (FI): **a**: EtOH, 90 °C, 24 h, 67%; **b**: POCl_3_, DMF, 60 °C, 78%; **c**: AcOH, CH_3_NO_2_, ammonium acetate, 65 °C, 64%; **d**: LiAlH_4_, THF, 80 °C, 90% (crude); **e**: NMP, DIPEA, microwave, 100–130 °C, 35%.

To develop the MOF dip-sensor, a model PET-fluoroionophore (FI) system was selected to enable the sensor to operate at a wavelength range compatible with low optical transmission loss in the lead-silicate (F2) glass from which the fibers are made (typical loss values for fibers made from this material are: 1.6 dB/m at 420 nm, 0.35 dB/m at 700 nm [24]). As the FI is excited by the intense guided light inside the MOF resulting from the highly confined core mode, it is important that the FI has reasonable photostability [9,25,26]. Due to the extensive utilization of naphthalimide fluorophores in fluorescent sensors [27], including commercial sensors, we selected a naphthalimide derived PET fluoroinonophore for this work. We used a simplified version of a sodium PET sensor previously synthesised by He *et al.* [21] as the basis of our MOF dip-sensor. The procedure used to synthesise FI is shown in Scheme 1. A commercially available ionophore, N-phenyl aza-15-crown-5 [28], was utilized which, using established literature procedures [21], can be linked to the photostable naphthalimide fluorophore via an ethyl spacer. Thus, FI possesses a similar structure to the PET sensor reported by He *et al.* [21], but for synthetic simplicity (16% overall yield, 4 linear steps) we omitted the methyl ether ortho to the aza-15-crown-5 and the carboxyl moiety that can be used for surface attachment. Synthetic procedures and characterization of the FI are described in the supplementary material.

### 2.2. Bulk (Cuvette) Fluorescence Measurements of the FI

FI and sodium perchlorate (98%) were dissolved in HPLC grade acetonitrile and ionic strength maintained with 100 mM tetraethylammonium perchlorate (NEt_4_ClO_4_), acting as an ionic buffer. Fluorescence spectra were recorded on a Varian Cary Eclipse spectrofluorometer. The excitation and emission slit width were set at 5 mm. All measurements were recorded at 25 °C using 700 μL quartz cuvettes with an excitation path-length of 10 mm. FI and sodium perchlorate solutions were pre-mixed and incubated at room temperature for at least 120 min before the spectra were recorded.

### 2.3. Experimental Setup for Measurement of PET Effect in Microstructured Optical Fibers

**Figure 1.**
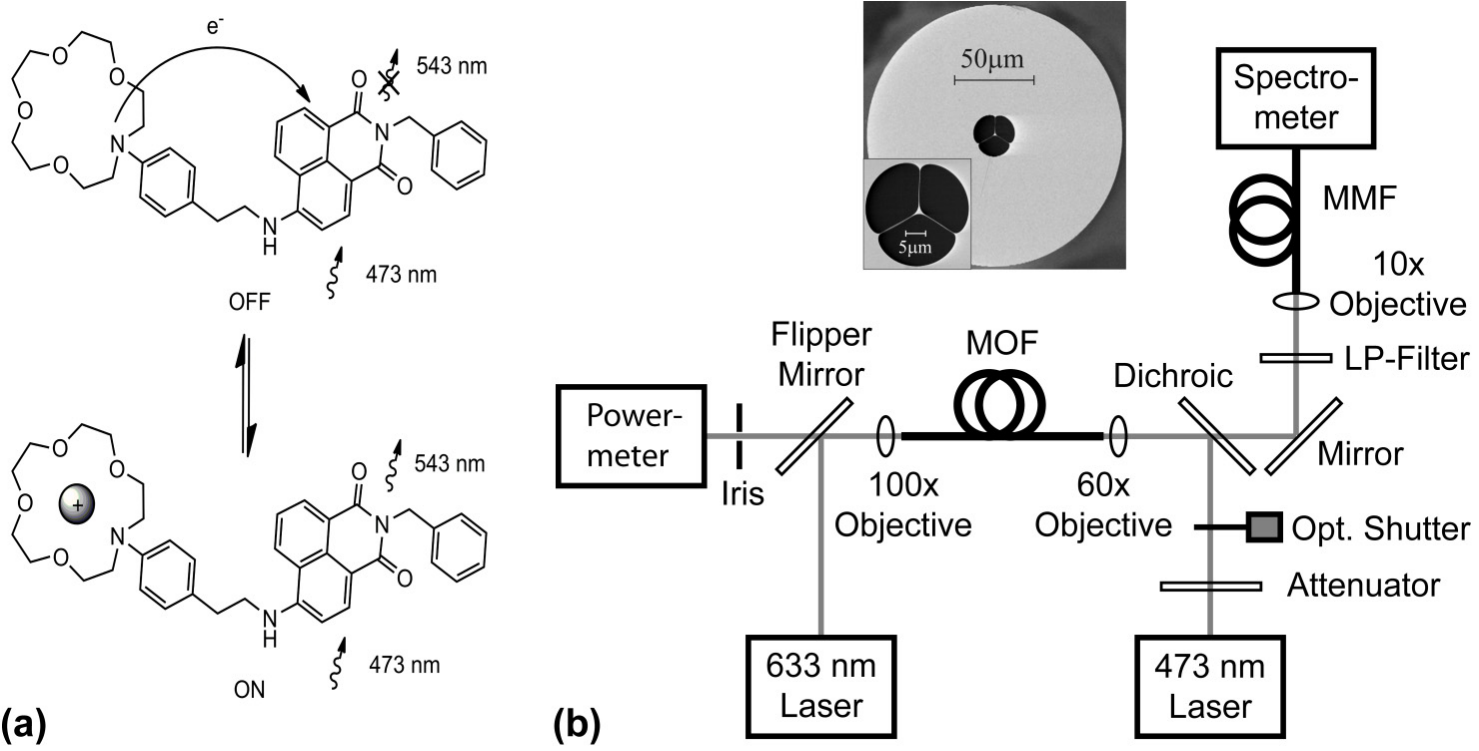
**(a)** ‘OFF-ON’ PET mechanism for cation sensing using the synthesized model PET-fluoroionophore (FI) system loaded into the holes of the fiber. **(b)** Experimental setup of the microstructured optical fiber dip-sensor used to measure the PET effect and photobleaching response. The MOF was filled by dipping its left-hand-side end (as per Figure 1(b)) into fluoroionophore solutions. The dichroic mirror and long-pass (LP) filter were used to separate the backward-propagating fluorescence and pump signals. The optical shutter minimizes unwanted photobleaching effects of the FI molecules inside the MOF holes and extends their life-time. A 633-nm laser was used for alignment and switched off during spectroscopic measurements. Inset shows a scanning electron microscope image of the MOF (F2, Schott glass, 1.3 μm dia. suspended-core, hole dia.: 10–15 μm, cladding dia.: 160 μm). The setup, measurement procedures and filling procedures are described in the supplementary material.

The proof-of-principle experimental setup for sensing sodium-ion solutions in MOFs based on the PET effect is depicted in Figure 1.

The FI molecules loaded in the holes of the MOF are excited by the portion of the guided mode from the 473-nm pump light located within the holes. A fraction of the excited fluorescent photons emitted are captured within the core and guided to the fiber ends, where forward- and backward-propagating fluorescence signals can be detected. A theoretical model describing this sensing principle was described in References [4,29]. This model enables predictions to be made of the measured fluorescence signal intensities at the fiber ends, which depend on the quantum efficiency of the fluorophore, the fiber design and the interaction length. In the experiments reported in this article, we detect backward-propagating fluorescence signals to utilize the left-hand-side fiber end as a dip-sensor.

First the background fluorescence response of the synthesized FI was measured. After free-space coupling optimization of the pump light into the fiber core, as described in the supplementary material, the holes of the fiber were partially filled with ∼0.1 μL of an analyte solution without sodium ions comprising only specific concentrations of ionic-buffer and FI molecules in a solvent (filling time: 3 min exactly). Background fluorescence spectra from 505–748 nm were recorded. Each spectrum is an average of 8 successively background fluorescence signal waveforms, each recorded with a pump beam exposure (*i.e.*, shutter open) of 2.5 × 10^−3^ s, resulting in an accumulated total exposure time of 0.02 s for the averaged spectrum. Thereafter, the corresponding PET fluorescence response was measured using a new 30-cm long piece of unfilled MOF, mounted between the two nanopositioning stages. Again, the free-space coupling into the MOF core was optimized. To ensure accurate comparison between background fluorescence and PET fluorescence spectra, care was taken to match the transmitted pump power to that of the previously used MOF as described in the supplementary material. To prove the principle of PET for sensing in microstructured fibers, this second piece of MOF was then partially filled with ∼0.1 μL of an analyte solution containing a specific concentration of sodium ions (*i.e.*, 925 ppm Na^+^ and 18.4 ppm Na^+^) but mixed with an identical solution of solvent, ionic-buffer and FI molecules as previously used. Using constant fiber lengths and filling-times ensured that this second piece of MOF contained a similar volume of analyte solution. After the filling process, PET fluorescence spectra were recorded, each spectrum consisting of an average of several signal waveforms.

## 3. Results and Discussion

### 3.1. Fluorescence Response of FI to Sodium Ions (in Cuvette)

**Figure 2.**
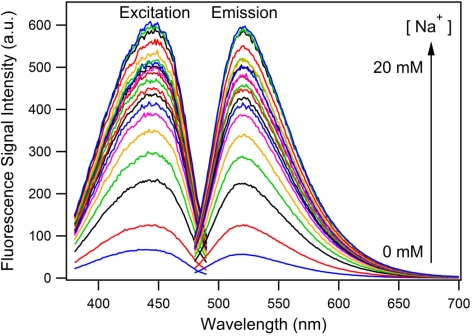
Excitation and emission spectra of the FI in acetonitrile ([NEt_4_ClO_4_] = 100 mM) with sodium ion (*i.e.*, sodium perchlorate) concentrations from 0–20 mM and constant FI concentration (0.02 mM). The excitation (left) and emission (right) spectra were recorded at fixed emission and excitation wavelengths of 520 nm and 440 nm, respectively.

As a consequence of the subtle changes made to the structure of FI to simplify the synthesis, we first sought to validate its operation as a PET sensor. As expected, Figure 2 shows that the intensity of the excitation and emission spectra both increase with the increasing concentration of sodium ions. No significant shift in the wavelength of the excitation or emission spectra occur as the concentration of sodium ions increased, showing that FI does not exhibit photoinduced charge transfer (PCT) and therefore is operating via a PET mechanism [30].

**Figure 3.**
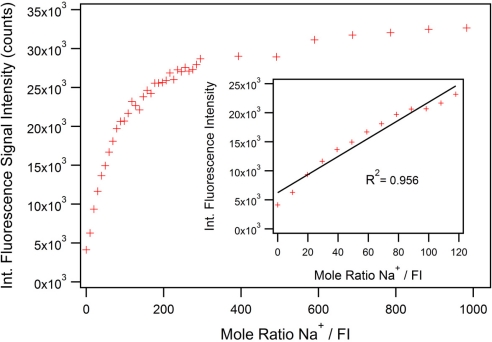
Integrated fluorescence intensity of different mole ratio of sodium to FI, recorded at an excitation wavelength of 470 nm. The inset shows the linear range of the PET effect including an applied linear fit with R^2^ = 0.956. A constant FI concentration of 0.02 mM was used; the concentration sodium ions was varied.

Simple aza-crowns bind alkali cations with binding constants in the molar range [31]; typically with stability constant (log K) ∼2 dm^3^ mol^−1^ in aqueous conditions [32] and ∼3 dm^3^ mol^−1^ in methanol [33,34]. These binding constants can be enhanced by providing additional donors [32] but this was not deemed necessary for our proof-of-concept dip-sensor. Figure 3 shows the integrated fluorescence intensity with respect to different ratios of sodium to FI. A clear saturation point cannot be identified before the sodium reaches 1,000 mole equivalence due to the relatively weak affinity of the aza-15-crown-5 ionophore for sodium ions [32]. The inset in Figure 3 shows the linear range (R^2^ = 0.956) of sodium detection is from 0 to *ca.* 120 mole equivalents in cuvette. As the FI cannot be fully quenched, even in a solution with no Na^+^, the linear fit cannot go through the origin. In all the MOF experiments (under high and low concentration regimes), the ratio of sodium to FI was kept at 100 to 1, within the linear range of sodium detection. Within this range, the fluorescence increases by a factor of up to 5.6.

### 3.2. Microstructured Optical Fiber Measurements

**Figure 4.**
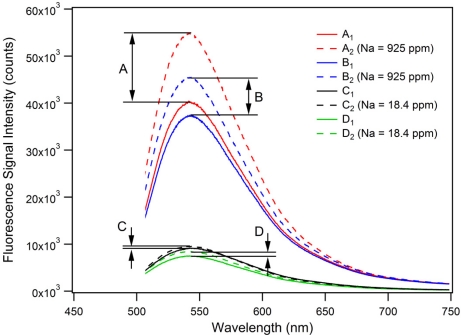
Fluorescence spectra of the PET-based MOF dip-sensors for analyte solutions, with and without sodium ions, showing the PET effect for small-volume (∼0.1 μL) ion detection. Traces A_1_ and B_1_ represent background fluorescence spectra, whereas the corresponding PET fluorescence spectra for Na^+^ = 925 ppm are shown as traces A_2_ and B_2_. For Na^+^ = 18.4 ppm, traces C_1_and D_1_ are the background fluorescence spectra, and traces C_2_ and D_2_ depict the corresponding PET-fluorescence spectra. Fluorescence signal intensity increases due to the PET effect: A = 36.4%, B = 21.4%, C = 4.1%, D = 12.2%.

The PET effect in microstructured optical fibers was investigated using the experimental setup and measurement procedure described in the supplementary material. Figure 4 shows the proof-of-principle PET detection using this fiber sensing architecture.

To show that the PET effect can be utilized in-fiber for high sodium ion concentrations, two sets of measurements (A and B) each containing a background fluorescence spectrum of FI ([FI] = 0.402 mM, [NEt_4_ClO_4_] = 100 mM in acetonitrile) as well as a subsequently acquired and corresponding PET fluorescence spectrum for a sodium-spiked sample ([Na^+^] = 40.2 mM (925 ppm), [FI] = 0.402 mM, [NEt_4_ClO_4_] = 100 mM in acetonitrile) were recorded. This high concentration of Na^+^ (925 ppm) was used to ensure that we were definitely operating in the saturation region of the FI, which can be seen to saturate at a ratio of 300 which corresponds to a sodium ion concentration of 6 mM (113 ppm) as seen in Figure 3. As Figure 4 shows, a fluorescence intensity increase, due to the PET effect, of A = 36.4% (peak-to-peak) with a signal-to-noise ratio (SNR) of 47.4 was measured. The second set of measurements contains the background fluorescence spectrum B_1_ (blue solid trace) and the sodium-spiked PET fluorescence spectrum B_2_, which was subsequently acquired (blue dashed trace in Figure 4). A resulting fluorescence signal intensity increase of B = 21.4% (peak-to-peak) with an SNR of 29.8 was determined.

These results demonstrate the feasibility of this PET-based small-volume ion detection approach. The difference of 15% in fluorescence signal increase between measurement sets A and B can be attributed to a combination of different sources of signal instabilities in the experimental setup and procedure. For example, free-space coupling instabilities of the pump beam into the MOF core translate to instabilities in the intensity levels for fluorophore excitation as well as fluorescence signal recapture. We expect to substantially improve the sensor performance by fusion splicing the soft-glass MOF to conventional silica fibers, and have reported initial fusion splicing results for these fibers with some success [35]. Such stable, permanently aligned connections with no exposed optical surfaces will improve the stability of the pump (excitation) and fluorescence signals which are launched in and out of the MOF, the sensors SNR, as well as prevent possible evaporation and crystallization of analyte solution at the fiber endface. Any variation in the hole diameters and filling times would also have affected sample volumes and therefore the intensity of the recorded spectra, though this is unlikely to account for the observed discrepancy. The differences in fluorescence enhancement due to the PET-effect for the bulk sample (5-fold as shown in Figure 3) compared to the MOF experiments (21.4–36.4% as in Figure 4) may be due to leaching of Na^+^ or K^+^ ions from the lead-silicate glass, resulting in a raised background fluorescence signal as the FI responds to the increased alkali cation concentration and consequently limiting the fluorescence enhancement when measuring spiked sodium solutions. Leaching under relatively benign conditions has been reported [36]. Future mitigation strategies include the use of coatings on the MOF surfaces or the use of alternative glass compositions.

To investigate the MOF-based PET sensor behavior for lower sodium ion concentrations, two additional sets of measurements (C and D) were recorded as represented in Figure 4. The first set of measurements contains the background fluorescence spectrum C_1_ (black solid trace) acquired using an un-spiked FI solution ([FI] = 0.0079 mM, [NEt_4_ClO_4_] = 100 mM in acetonitrile). The corresponding PET fluorescence spectrum C_2_ (black dashed trace) was subsequently acquired using a sodium-spiked FI solution ([Na^+^] = 0.799 mM (18.4 ppm), [FI] = 0.0079 mM, [NEt_4_ClO_4_] = 100 mM in acetonitrile). The second set of measurements shows the background fluorescence spectrum D_1_ (green solid trace) and the corresponding PET fluorescence spectrum D_2_ (green dashed trace). For measurement set C, a resulting fluorescence signal intensity increase of 4.1% (peak-to-peak) with an SNR of 3.9 was calculated, whereas for measurement set D a fluorescence signal intensity increase of 12.2% with an SNR of 10.1 was determined. Again, the difference of 8.1% in fluorescence signal increase between both sets of measurements (C and D) can be attributed to instabilities in the experimental setup. The low-concentration measurements (C and D) are close to the detection limit of the current MOF-based PET-sensor, and show the potential of this technique for small-volume ion-detection in MOFs.

Between the bulk-sample experiments (Figure 2) and the MOF measurements depicted in Figure 4, we noticed an intriguing 21 nm wavelength shift in the emission maxima of the fluorescence spectra (522 nm *vs.* 543 nm). The wavelength calibration of the two spectrometers was verified at several wavelengths and fluorophores. We observed a similar difference between bulk measurements for Lucifer Yellow but not Rhodamine 6G. Due to the structural similarities between the FI reported in this work and Lucifer Yellow we consider this wavelength shift may be related to the naphthalimide core of these two fluorophores. This is the focus of ongoing investigations.

An important consideration, for the successful development of PET-based MOF dip-sensors, is the photostability and photobleaching response of the FI inside the MOF. In our experience [26], organic fluorophores exhibit strong photobleaching behavior in the small-volume high intensity MOF environment. When exposed to the intense and locally concentrated light inside a suspended-core MOF, the life-time of such fluorophores typically only extends to a couple of minutes under constant illumination. To avoid significant photobleaching, the FI molecules were only excited during acquisition of each fluorescence spectrum. At all other times, the optical shutter blocks interaction with the 473-nm pump radiation, thereby extending the life-time of the fluorophore.

**Figure 5.**
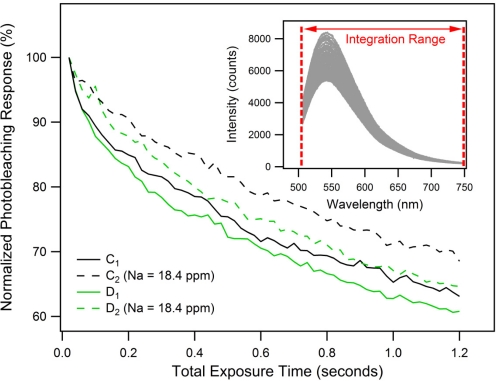
Normalized photobleaching response of the PET-based MOF dip sensor. Traces C_1_ and D_1_ show the background photobleaching responses of the analyte solution without sodium ions; traces C_2_ and D_2_ depict the corresponding PET-fluorescence responses of the analyte solution spiked with sodium ions (Na^+^ = 18.4 ppm). The inset shows the series of 60 successively acquired fluorescence spectra (each an average of 8 signal waveforms with a total pump beam exposure time of 0.02 s), integrated from 505–748 nm and normalized to generate trace D_2_ of Figure 5.

To investigate the photostability and photobleaching response [25] of the FI molecule, 60 successively recorded fluorescence spectra were obtained. Again, each spectrum was an average of 8 signal waveforms, recorded with a total exposure time of 0.02 s. Background fluorescence spectra C_1_ and D_1_, as well as the corresponding PET fluorescence spectra C_2_ and D_2_ shown in Figure 4, were the first from each individual series of 60 successively recorded spectra. Every spectrum from each series was integrated over the wavelength range of 505–748 nm and then normalized to obtain the photobleaching responses characterized by the graph in Figure 5.

The results show that after acquisition of 60 data points with an accumulated total exposure time of 1.2 s, the fluorescence intensity decays to 60–70% of its original value. The difference in fluorescence decays between measurement sets C and D can again be attributed to signal instabilities as described above. The un-spiked FI samples C_1_ and D_1_ exhibit 4–5.5% faster fluorescence decays compared to the sodium-spiked samples C_2_ and D_2_. This greater rate of decay is likely to be a consequence of the trapped excited state that occurs for the FI. These results confirm that our model FI system is suitable for use in small-volume, suspended-core MOF dip-sensors provided that a shutter is used.

The simple ionophore used in FI has limited selectivity for alkali cations, as mentioned in Section 3.1. We have identified two approaches for providing ion selectivity; the use of ion-selective membranes [37], which can be attached to the tip of the fiber prior to immersion in the analyte solution, and use of cryptand-based ionophores that can confer better ion selectivity. We are currently developing ion-selective FI molecules that can be surface-functionalized to the inner glass surface of the fiber holes and enable ion-selective sensing in aqueous solutions.

Ultimately this research will lead to optical fiber dip-sensors that combine the advantages of MOFs with the benefits of the fluorescence PET effect for biological, chemical and environmental sensing applications. Such composite sensing architectures have the potential to offer new, low cost, rugged and flexible sensor platforms that are field-deployable. This would eliminate the need for expensive and time consuming laboratory-based measurements based on ion-selective electrodes [11]. Also, it should be possible to use surface-attached ion-selective PET-fluoroionophores [38] to customize the fusion-spliced fiber dip sensing probes for the detection of different ion species, multiple-ion detection or even multiplexing. We are also evaluating different sensing strategies such as time-resolved lanthanide probes which might be applied in the future to this PET-based sensing scheme.

## 4. Conclusions

This paper presents the first PET-based microstructured optical fiber dip-sensor for small-volume ion sensing. Proof-of-principle was demonstrated by detection of sodium ion solutions down to 18.4 ppm, employing a soft-glass suspended-core optical fiber combined with a synthesized model PET-fluoroionophore system that was demonstrated to be suitable for use in intense locally concentrated light fields. The sensor performance indicates strong potential for the development of a cost-effective, flexible and portable sensor platform. Future research will seek to improve the signal stability, sensitivity and SNR. The introduction of an ion-selective version of this dip-sensor, as well as the use of PET-fluoroionophore surface attachment strategies for “real-world” aqueous-based sample detection are both underway.

## Supplementary Information
