# Supplementary material for: Photoinduced Electron Transfer Based Ion Sensing within an Optical Fiber
Source: Sensors (Basel). 2011 Oct 11;11(10):9560–72. doi: 10.3390/s111009560 (PMC3231295; doi:10.3390/s111009560)
Supplement: Supplementary file 1 [file sensors-11-09560-s001.pdf]

Supplementary Information

Photoinduced Electron Transfer Based Ion Sensing within an Optical Fiber

Florian V. Englich *, Tze Cheung Foo, Andrew C. Richardson, Heike Ebendorff-Heidepriem, Christopher J. Sumby and Tanya M. Monro

Institute for Photonics & Advanced Sensing (IPAS) and School of Chemistry & Physics, The University of Adelaide, North Terrace, SA 5005, Australia;
E-Mails: tze.foo@adelaide.edu.au (T.C.F.); andrew.richardson@adelaide.edu.au (A.C.R.); heike.ebendorff@adelaide.edu.au (H.E.-H.); christopher.sumby@adelaide.edu.au (C.J.S.); tanya.monro@adelaide.edu.au (T.M.M.)

* Author to whom correspondence should be addressed; E-Mail: florian.englich@adelaide.edu.au; Tel.: +61-8831-30816; Fax: +61-8830-34380.

Received: 8 September 2011; in revised form: 5 October 2011 / Accepted: 7 October 2011 / Published: 11 October 2011

---

1. General Experimental Procedures for Synthesis

All commercially available reagents were used without further purification. Thin layer chromatograms were run on MERCK aluminum-backed silica gel 60 F_{254} plates (20 × 20 cm, 0.25 mm thickness) and viewed under 254 nm UV light. Flash column chromatography was performed using Davisil silica gel 60 (particle size 0.040–0.063 mm) from Grace Gmbh and Co. KG following the guidelines outlined in literature [1]. Microwave synthesis was performed in the CEM focused microwave^{TM} synthesis system, (Discover@ SP) using a reaction tube and seal provided by CEM. Microwave irradiation started after 30 seconds pre-mixing. The microwave power was variable but the set point temperature was fixed.

^{1}H and ^{13}C NMR spectra were obtained using either a Varian Gemini 2000 Spectrometer (^{1}H: 300.13 MHz, ^{13}C: 75.48) or a Varian Inova Spectrometer (^{1}H: 599.842, ^{13}C: 150.842 MHz). The chemical values are given on δ scale quoted in parts per million (ppm), followed by the integration, multiplicity, coupling constant J and assignment. All spectra were recorded in chloroform-D from Cambridge Isotope Laboratories. ^{1}H NMR spectra recorded in $CDCl_3$ were referenced relative to the internal standard Me_4Si, $\delta_H = 0.0$, while for ^{13}C NMR spectra using $CDCl_3$ used $\delta_C = 77.23$ as an internal standard. The following abbreviations for proton multiplicities are used: s, singlet; d, doublet; t, triplet; br, broad singlet; m, multiplet; indicates a broadened signal due to unresolved J value(s). ^{1}H and ^{13}C NMR signals for compound **7** and **FI** were assigned by the combination of information from Correlation spectroscopy (COSY), Nuclear Overhauser effect spectroscopy (NOESY), Heteronuclear Single Quantum Coherence (HSQC) and Heteronuclear Multiple Bond Correlation (HMBC) performed on the Varian Inova Spectrometer (^{1}H: 599.842, ^{13}C: 150.842 MHz).

All infrared spectra were obtained using a Perkin Elmer Spectrum 100 UATR FT-IR spectrometer mounted with a diamond coated zinc selenide crystal. All electrospray ionization (ESI) mass spectra, except for compound **3**, were obtained using a Finnigan LCQ mass spectrometer. The electron impact (EI) mass spectrum of compound **3** was obtained on a Shimadzu GCMS-QP5050A mass spectrometer. Samples are all diluted with HPLC grade methanol. High resolution mass spectrometry (HRMS) was performed by the Adelaide Proteomics Centre, The University of Adelaide, using LTQ Orbitrap XL mass spectrometer from Thermo Fisher Scientific. All melting points were determined by Gallenkamp variable heat melting point apparatus.

1.1. Synthesis of FI

N-Benzyl-4-chloro-1,8-naphthalimide (3): 4-Chloro-1,8-naphthalic anhydride (2.00 g, 8.60 mmol) was stirred in 30 mL ethanol at 90 °C for 20 min under nitrogen atmosphere. Benzyl amine (950 μL, 8.70 mmol) was added into the reaction mixture and the resulting solution was heated at reflux for 66 h. The crude product was collected by filtration, washed with ethanol and dried under vacuum. The crude product was recrystallised from ethanol to afford **3** as yellow crystals (1.84 g, 67%). m.p. 175–177 °C; ^{1}H NMR (300 MHz, $CDCl_3$) d = 8.68 (d, *J* = 6.6 Hz, 1 H), 8.61 (d, *J* = 8.8 Hz, 1 H), 8.52 (d, *J* = 7.7 Hz, 1 H), 7.90–7.80 (m, 2 H), 7.58–7.49 (m, 2 H), 7.35–7.19 (m, 3 H), 5.38 (s, 2 H); UATR FTIR (Diamond/ZnSe) υ/cm^{-1} 3,065, 1,916, 1,698 and 1,655; MS (EI, +ve mode) *m/z* 321 ([M-H]$^+$), 215, 187, 126, 91, 77, 65, 51.

4-Formyl-phenyl-15-crown-5 (5): Based on the method of He [2], phenyl-15-crown-5 (1.98 g, 6.70 mmol) was dissolved in dimethylformamide (DMF) (33 mL), cooled to −4 °C, and phosphoryl chloride ($POCl_3$) (1.2 mL, 13.1 mmol) added to the mixture via precision syringe. The reaction mixture was stirred at *ca.* 0 °C for 20 min, then at room temperature overnight before heating up to 60 °C for 1 h. After cooling to room temperature the reaction mixture was poured into water (100 mL), the residue remaining in the flask rinsed with water (40 mL), and the pH of the combined aqueous solutions adjusted to pH 7 using saturated aqueous potassium carbonate solutions. The aqueous solution was extracted with ethyl acetate (4 × 100 mL), the extracts dried with magnesium sulfate, the solvent removed under vacuum, and the resulting residue dried under high vacuum at ~50 °C for 10 to 12 h to afford **5** as a pale brown colored solid (1.66 g, 78%). m.p. 87–89 °C; ^{1}H NMR (300 MHz, $CDCl_3$) δ 9.73 (1 H, s) 7.72 (2 H, d, J = 9 Hz), 6.71 (2 H, d, J = 9 Hz), 3.59–3.83 (20 H, m); UATR FTIR (Diamond/ZnSe) υ/cm^{-1}, 2,869, 2,797, 2,720, 2,697, 1,600, 1,591, 1,553 and 1,524; MS (ESI, +ve mode) *m/z* 347.2 (17%), 346.1 ([M+Na]$^+$, 100%).

4-Nitroethylenyl-phenylaza-15-crown-5 (6): Compound **5** (1.66 g, 5.13 mmol) and ammonium acetate (4.06 g, 52.7 mmol) were stirred in glacial acetic acid (10 mL) and nitromethane (6.5 mL) at room temperature for 10 min before being heated at 65 °C for 5 h. After cooling, the reaction mixture was diluted with chloroform (20 mL), rinsed with water (3 × 50 mL) and the aqueous washings re-extracted with further chloroform (3 × 50 mL). The chlorinated extracts were combined, dried with magnesium sulfate and the solvent removed in vacuum. The crude product was dried under high vacuum overnight and then recrystallised from ethyl acetate/hexane (~1:3) to afford compound **6** as red needle-shaped crystal (1.20 g, 64%). m.p. 131–133 °C; ^{1}H NMR (600 MHz, $CDCl_3$) d = 7.95 (d, *J* = 13.5 Hz, 1 H, H23), 7.49 (d, *J* = 13.5 Hz, 1 H, H22), 7.40 (d, *J* = 8.8 Hz, 2 H, H18, H20), 6.68

(d, J = 8.8 Hz, 2 H, H17, H21), 3.81–3.60 (m, 20 H, CH_2 from aza-15-crown-5); ^{13}C NMR (151 MHz, $CDCl_3$) d = 151.25(C16), 140.34(C23), 132.34(C22), 131.81 (C20, C18), 117.55 (C19), 112.16 (C17, C21), 71.52, 70.53, 70.23, 68.40, 53.06 (5 × C from aza-15-crown-5); UATR FTIR (Diamond/ZnSe) υ/cm^{-1} 2,866, 1,593 and 1,527; MS (ESI, +ve mode) *m/z* 390.1 (21%), 389 ($[M+Na]^+$, 100%); HRMS (ESI) *m/z* found 367.18672, $[C_{18}H_{26}N_2O_6 + H]^+$ required 367.18636.

4-Aminoethyl-phenylaza-15-crown-5 (7): Lithium aluminum hydride (1.45 g, 38.2 mmol) was suspended in freshly distilled tetrahydrofuran (THF) (50 mL) at room temperature. Compound **6** (699 mg, 1.91 mmol) was dissolved in THF (50 mL) and added dropwise to the reaction mixture. The reaction mixture was heated to reflux for 5 h. The reaction was quenched by addition of potassium hydroxide solution (6 M, 47 mL). Further THF (47 mL) was added to the mixture, the slurry filtered and the solid pellet further washed with THF (4 × 25 mL). Two phases were separated from the supernatant. THF was removed and the residue was redissolved in chloroform (50 mL). The chloroform solution was raised with water (2 × 50 mL) and the aqueous washings were combined with the potassium hydroxide solution separated from the supernatant. The combined basic aqueous solution was extracted with chloroform (2 × 50 mL). All chloroform solutions were then combined and dried with magnesium sulfate. The solvent was removed under vacuum to give **7** as an orange-brown oil (580 mg, 90%). The product was used in the next step without further purification. ^1H NMR (600 MHz, $CDCl_3$) d 7.03 (d, J = 8.2 Hz, 2 H, H20, H18), 6.61 (d, J = 8.8 Hz, 2 H, H21, H17), 3.80–3.51 (m, 30 H, H from aza-15-crown-5), 2.90 (t, J = 7.0 Hz, 2 H, H22), 2.63 (t, J = 7.0 Hz, 2 H, H23), 1.55 (br, NH_2); ^{13}C NMR (151 MHz, $CDCl_3$) d 146.30 (C16), 129.87 (C20, C18), 126.96 (C19), 111.72 (C21, C17), 71.55, 70.41, 68.93, 52.72 (5 × C from aza-15-crown-5 with one overlapping peak), 44.05 (C22) 39.15 (C23); UATR FTIR (Diamond/ZnSe) υ/cm^{-1} 2,862, 1,615 and 1,518; MS (ESI, +ve mode) *m/z* 405.1 (20%), 361.2 ($[M+Na]^+$, 100%), 322.2 (24%); HRMS (ESI) *m/z* found 339.22785, $[C_{18}H_{30}N_2O_4+H]^+$ required 339.22783.

N-Benzyl-[4-(aza-15-crown-5)-phenylethylamino]-1,8-naphthalimide (FI): Compound **3** (199 mg, 0.618 mmol), **7** (418 mg, 1.24 mmol) and *N*,*N*-diisopropylethylamine (DIPEA) (272 µL, 1.56 mmol) were dissolved in *N*-methyl-2-pyrrolidone (NMP) (18 mL) in a CEM ™ reaction tube. The tube was sealed and heated at 100 °C under microwave irradiation for 4 h and then at 130 °C under microwave irradiation for 1 h (the reaction progress was monitored by TLC at hourly intervals after the tube was cooled down to 60 °C). The NMP was removed by distillation under reduced pressure at 50–60 °C and the crude product was dried at ~50 °C under high vacuum. Purification on silica gel with 1:1 ethyl acetate:chloroform eluate afforded **FI** as orange crystals (136 mg, 35%). m.p. 149–152 °C; ^1H NMR (600 MHz ,$CDCl_3$) d = 8.57 (d, J = 6.4 Hz, 1 H, H33), 8.48 (d, J = 8.2 Hz, 1 H, H27), 7.91 (d, J = 8.2 Hz, 1 H, H35), 7.57 (t, J = 7.9 Hz, 1H, H34), 7.35 (d, J = 7.0 Hz, 1H, H41, H45) 7.31–7.18 (m, 3 H, H42, H43, H44), 7.10 (d, J = 8.2 Hz, 2 H, H18, H20), 6.76 (d, J = 8.2 Hz, 1 H, H26), 6.65 (d, J = 8.2 Hz, 2 H, H17, H21), 5.36 (s, 2 H, H39 × 2), 5.31 (br. s., 1 H, NH), 3.81–3.54 (m, 22 H, CH_2 from aza-15-crown-5 and H23), 2.98 (t, J = 6.7 Hz, 2 H, H22 × 2); ^{13}C NMR (151 MHz,$CDCl_3$) d 164.92 (C32), 164.33 (C29, C31), 149.60 (C25), 146.89 (C16), 138.07 (C40), 134.94 (C27), 131.51 (C33), 130.12 (C37), 129.79 (C18), C20), 129.03 (C41 and C45), 128.54 (C42, C43 and C44), 127.39 (C42, C43 and C44), 126.17 (C35), 124.92 (C34), 124.86 (C19), 123.33 (C40), 120.54 (C36), 112.06 (C17, C21), 110.48 (C25), 104.76 (C26), 71.55, 70.43, 70.29, 68.81, 68.37, 52.75, 44.91 (C23 and 5 × CH_2 from aza-15-crown-5), 43.52 (C39), 33.89 (C22); UATR FTIR

(Diamond/ZnSe) υ/cm^{-1} 3,364, 2,863, 1,683, 1,636, 1,616, 1,573, 1,545 and 1,518; MS (ESI, +ve mode) *m/z* 646.3 ($[M+Na]^+$, 100%), 643.2 (35%), 624.2 (29%), 515.0 (30%), 415.9 (30%); HRMS (ESI) *m/z* found 624.30748, $[C_{37}H_{41}N_3O_6+H]^+$ required 624.30681.

1.2. Physical Measurements

Tetraethylammonium perchlorate (NEt_4ClO_4) was prepared as described [3] (Caution! Perchlorate salts are strong oxidizing agents and are potentially explosive and should be handled with care).

A matched pair 700 µL quartz cuvettes from Starna with excitation path of 10 mm were used in the fluorescence emission measurement. The temperature during the fluorescence measurement was controlled using a Peltier Thermostatted Multicell Holder Accessory fitted to the Varian Cary Eclipse spectrofluorometer.

2. Microstructured Optical Fiber Fabrication

For the fabrication of the microstructured optical fiber, we used commercial F2 lead silicate glass (Schott Glass Co.) [4]. The fiber was made using a three step process. Firstly, an air/glass structured preform and a jacket tube were manufactured from F2 glass billets using the extrusion technique [5]. The outer diameter of these extruded items was 10 mm. The inner diameter of the tube was 1 mm. In the second step, the preform was reduced in size to a "cane" of ~0.7 mm outer diameter using a fiber drawing tower. Next the cane was inserted into the jacket tube. The diameter of the cane was selected to provide a gap of ~0.3 mm between the cane and the hole of the tube. This gap was chosen to allow active inflation of the holes during the drawing of the cane-in-tube assembly. This assembly was finally drawn down to form the microstructured optical fiber. Careful adjustment of the drawing speed allowed accurate control of the outer diameter of the fiber to 160 µm within ±1 µm. At the beginning of the fiber drawing, we gradually increased the pressure inside the air holes of the cane. When reaching 4.8 kPa, the holes size was increased to such an extent that the outer wall of the cane fused with the inner wall of the tube. From that stage in the fiber draw, this air hole pressure was maintained and 70 m of fiber with consistent cross-sectional structure was drawn.

Figure S1. Scanning electron microscope images of **(a)** fiber drawn without hole pressurization, and **(b)** fiber drawn using 4.8 kPa hole pressurization.

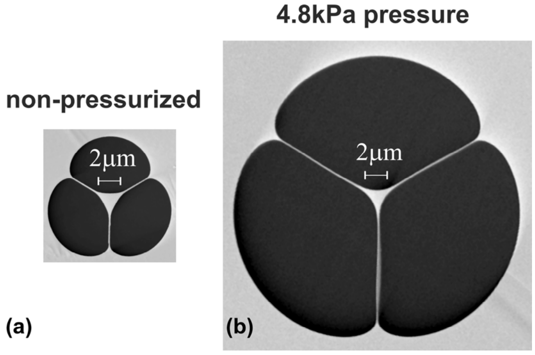

We used active hole pressurization in order to increase the hole diameter and thus enhance liquid filling rate and reduce hole blockages. Using electron microscope images of the fiber cross-section, the

core diameter and approximate air hole diameter were measured to be 1.2 µm and ~13 µm. Compare, the hole diameter of fibers drawn using no active pressurization is ~6 µm for the same core diameter of 1.2 µm, as depicted in Figure S1.

3. Microstructured Optical Fiber Dip-Sensor Setup and Measurement Procedures

A diode-pumped, solid-state laser at 473 nm (CrystaLaser CL-473-025, continuous wave, TEM_{00}, linearly polarized, 25 mW) was used as an excitation source once the FI solutions had been filled into the holes of the microstructured optical fiber. The excitation beam was attenuated to 2.5 mW using a neutral-density filter and free-space coupled, via a dichroic mirror (HR: 471–491 nm, edge-λ: 497 nm, HT: 503.3–900 nm), into a microscope objective (Edmund optics 60×, NA: 0.75) where it was focused into the suspended-core of the MOF (RHS end of Figure 1). The transmitted pump signal was collected by a second microscope objective (Edmund optics 100×, NA: 1.25), as shown on the LHS of Figure 1, and focused onto the power meter. To ensure accurate control of the light coupled in and out of the fiber core, both ends of the MOF were mounted onto two separate 3-axis nanopositioning stages (Thorlabs Model MAX316). A free-space coupling efficiency of 10–15% of pump light into the fiber core was achieved. An attenuation of ~2 dB/m at 473 nm for the unfilled fiber was measured, using the standard cutback-method.

After collimation by the 60× objective lens, the backward-propagating fluorescence signal was separated from the back coupled pump beam using the dichroic mirror. Furthermore, a long-pass filter (pass band: 504.7–900 nm) was used to block any residual pump radiation leaking through the dichroic mirror. The pure backward-propagating fluorescence signal was then coupled via a microscope objective (Olympus 10×, NA: 0.25) into a multimode fiber (Thorlabs, core dia.: 400 µm, NA: 0.48), connected to an imaging spectrometer (Horiba Jobin Yvon iHR320) with cooled 1,024 × 256 pixel CCD camera as per Figure 1. An optical shutter synchronized with the data-acquisition routine of the spectrometer is used to shield the FI molecules inside the fiber holes from the 473-nm pump radiation between acquisitions of fluorescence spectra. This minimized unwanted photobleaching effects, therefore, prolonging the fluorescence life-time of the FI molecules.

Optimization of the in coupled pump beam was achieved using the nanopositioning stage to position the fiber end at the focus of the 60× objective and by monitoring the transmitted pump power coupled onto the optical power meter, as shown in Figure 1. The power meter and the nanopositioning stage containing the 100× objective were also carefully aligned to efficiently detect only the pump light exiting the fiber core. An iris was placed in front of the power meter to block-out residual transmitted cladding light. A maximum transmitted power of ~270–360 µW through the unfilled MOFs was measured.

To increase the long-term free-space coupling stability of the pump beam into the MOF core, the 60× objective end of the MOF was slightly defocused from the objective using the nanopositioning stage, thus enlarging the pump beam diameter covering the 1.3 µm suspended-core endface. By always adjusting the transmitted power through the unfilled fiber to ~200 µW, this procedure ensured reproducibility of the transmitted pump power and enabled comparisons of spectra from different pieces of MOF. To optimize the alignment of the backward-propagating fluorescence signal into the

spectrometer, a strongly attenuated HeNe laser at 633 nm was coupled into the core of the MOF from the 100× objective end and then switched off prior to fluorescence measurements.

In our experiment, we used 30-cm long pieces of suspended-core MOF (core diameter: 1.3 µm, inflated holes with diameter: 10–15 µm, cladding diameter: 160 µm) with cleaved ends. First, an unfilled MOF was mounted between the 3-axis nanopositioning stages and its pump light transmission and fluorescence signals were optimized as described above. Then, the 100× objective end of the MOF, Figure 1, was carefully removed from its 3-axis nanopositioning stage and dipped into the analyte solution. By capillary forces, the holes in the MOF were filled with the analyte solution for exactly 3 min. After this time the 100× objective end of the fiber was immediately removed from the analyte solution and secured back onto its nanopositioning stage. This resulted in a partially filled (*i.e.*, 20 cm) microstructured fiber with ~0.1 µL analyte solution inside the holes of the MOF, assuming a fiber hole diameter of 15 µm. By partially filling the fiber holes, no analyte solution was able to evaporate at the 60× objective endface of the MOF, used for free-space coupling the pump (excitation) beam into the fiber core. Evaporation of the analyte solution containing ionic buffer, tetraethylammonium perchlorate, was found to be undesirable because it led to crystallization at the fiber endface and thus prevented reproducible coupling of light in and out of the fiber.

4. Selected ^{1}H and ^{13}C NMR Spectra

Figure S2. 300 MHz ^{1}H NMR spectrum of *N*-benzyl-4-chloro-1,8-naphthalimide (**3**) in $CDCl_3$.

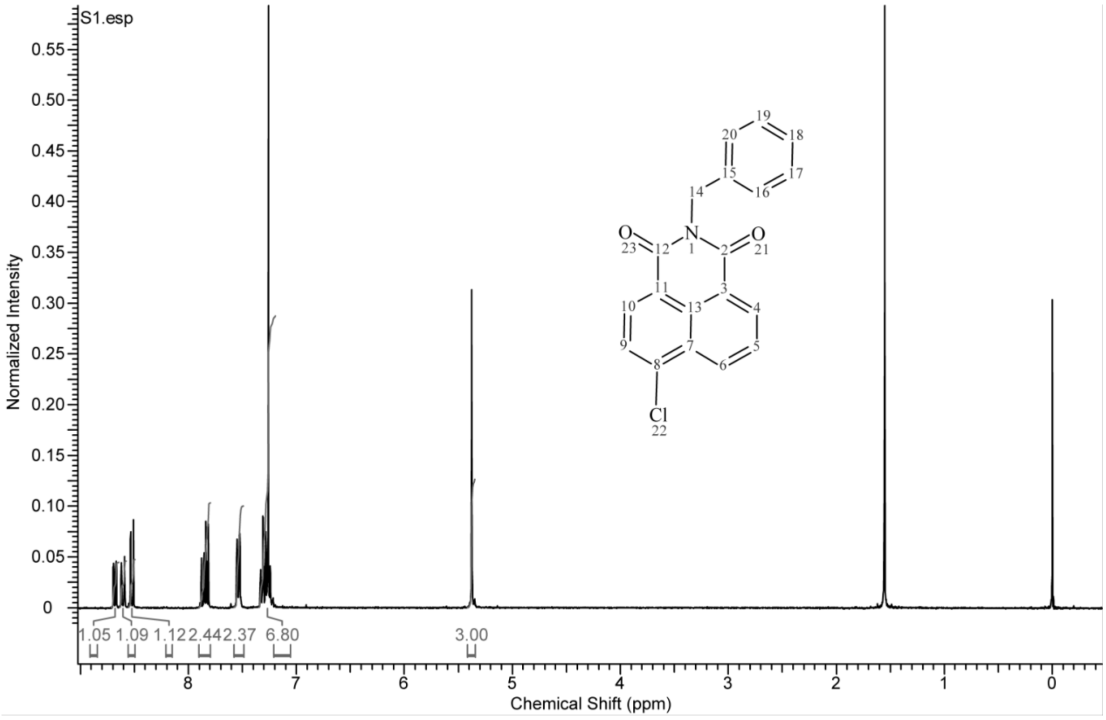

Figure S3. 300 MHz ^{1}H NMR spectrum of 4-formyl-phenyl-15-crown-5 (**5**) in $CDCl_3$.

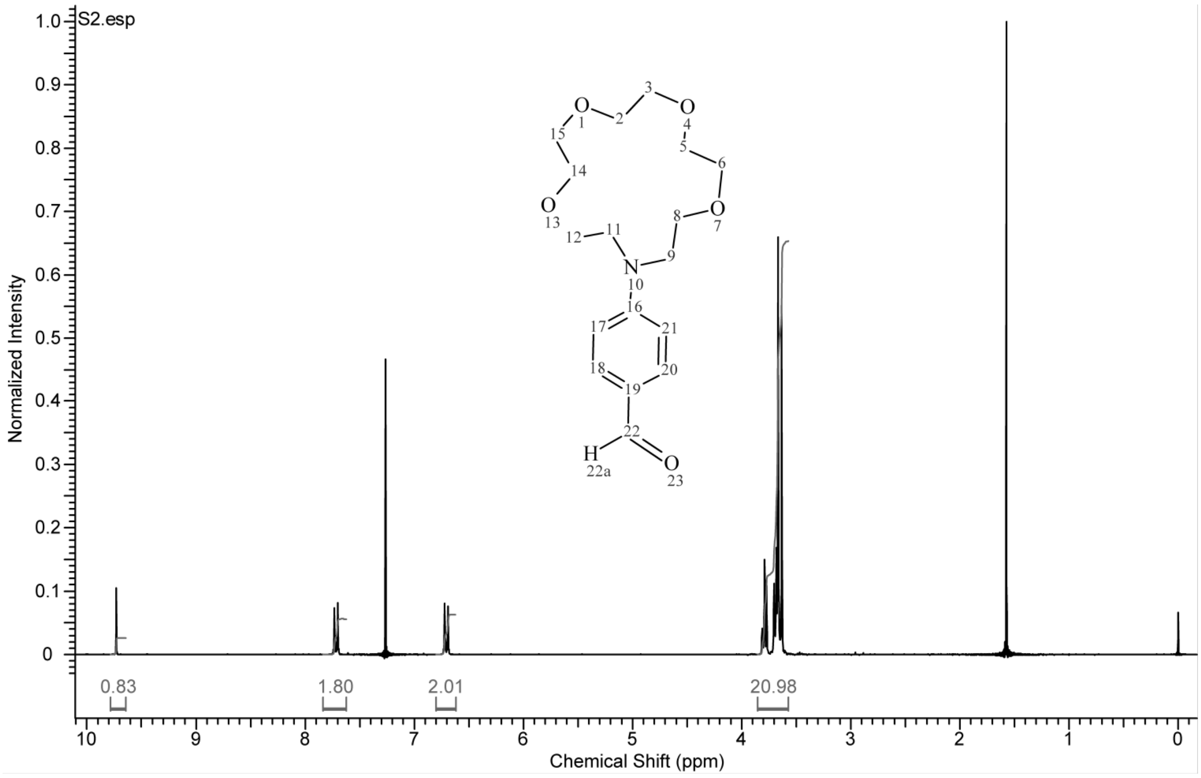

Figure S4. 600 MHz ^{1}H NMR spectrum of 4-nitroethylenyl-phenylaza-15-crown-5 (**6**).

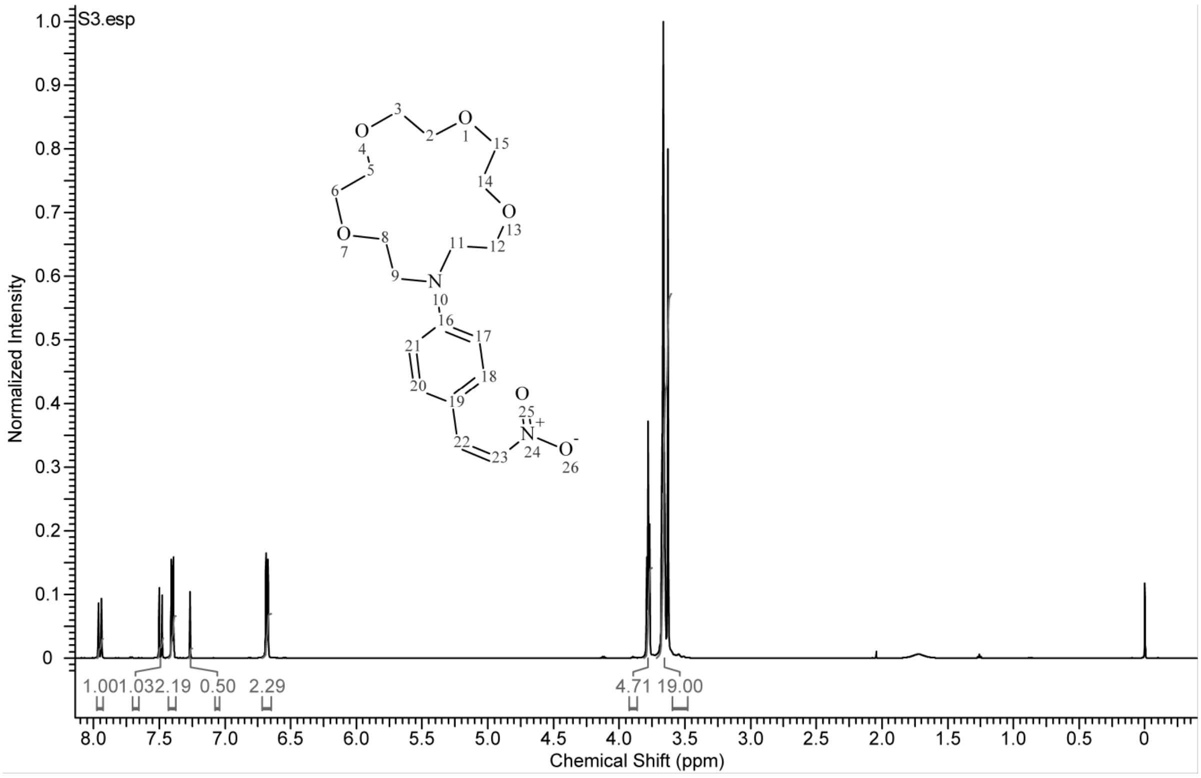

Figure S5. 151 MHz ^{13}C NMR spectrum of 4-nitroethylenyl-phenylaza-15-crown-5 (**6**).

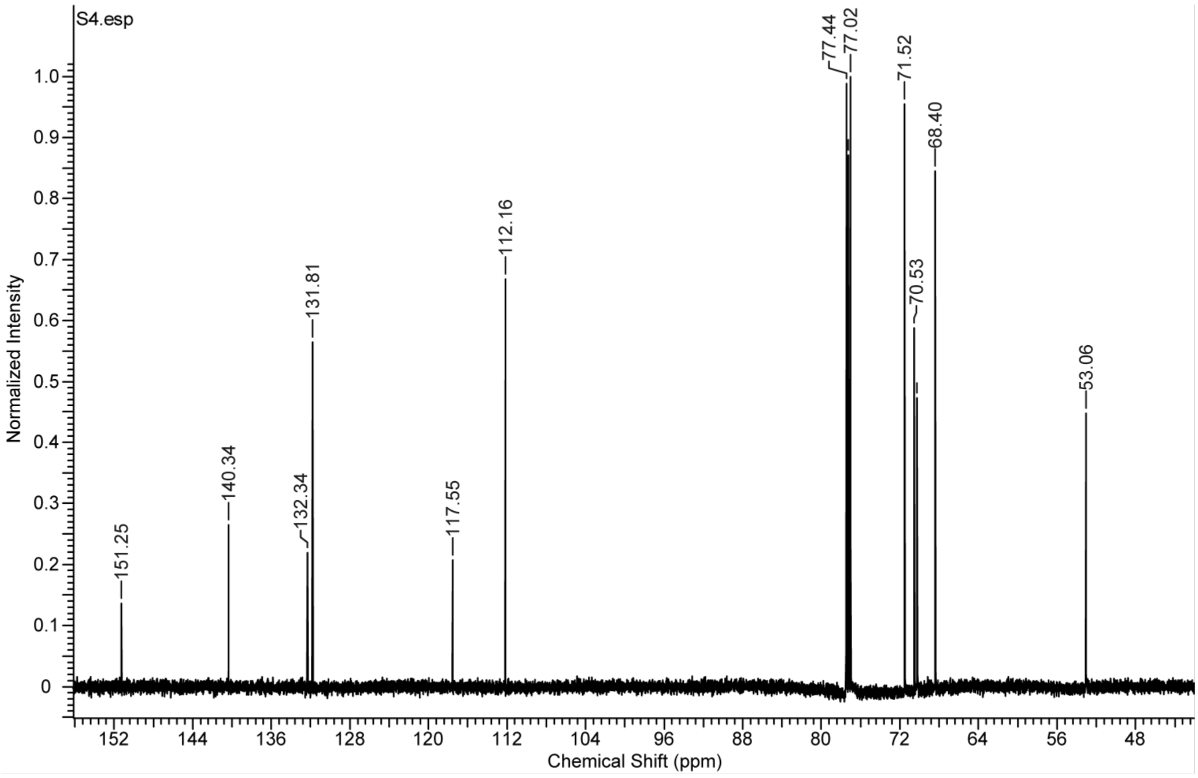

Figure S6. 600 MHz ^{1}H NMR spectrum of 4-aminoethyl-phenylaza-15-crown-5 (**7**).

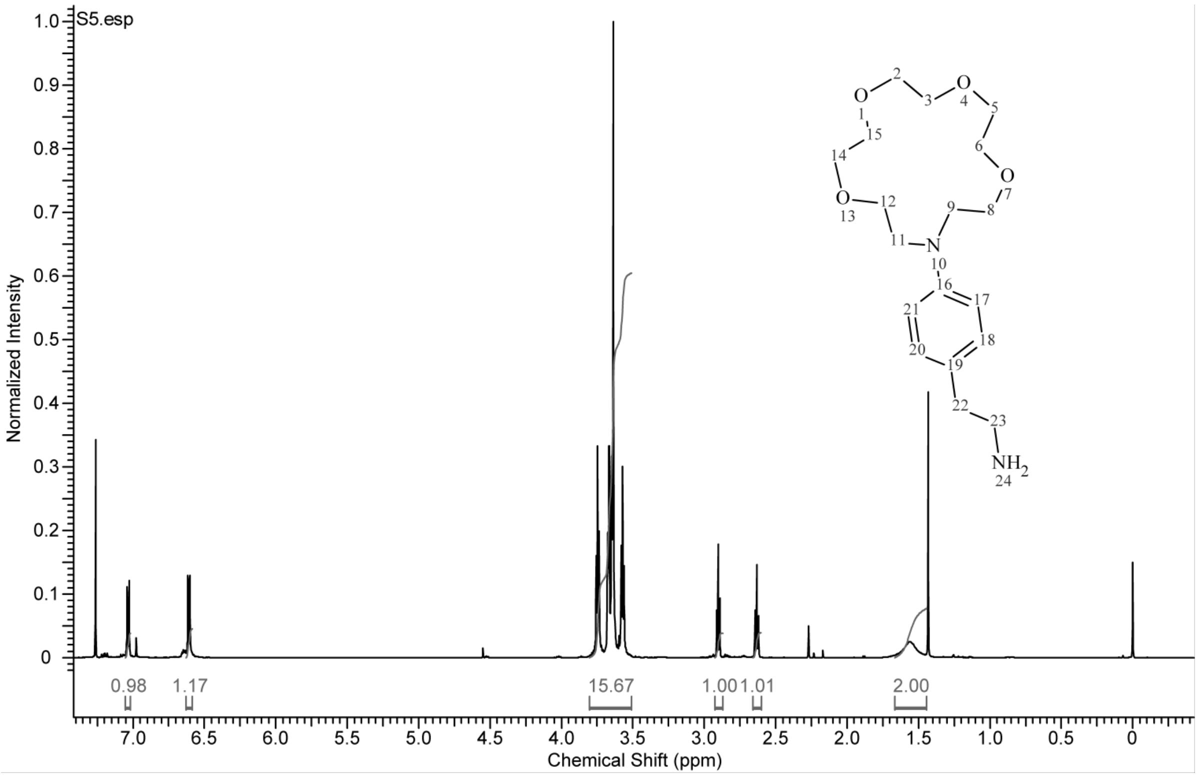

Figure S7. 151 MHz ^{13}C NMR spectrum of 4-aminoethyl-phenylaza-15-crown-5 (**7**).

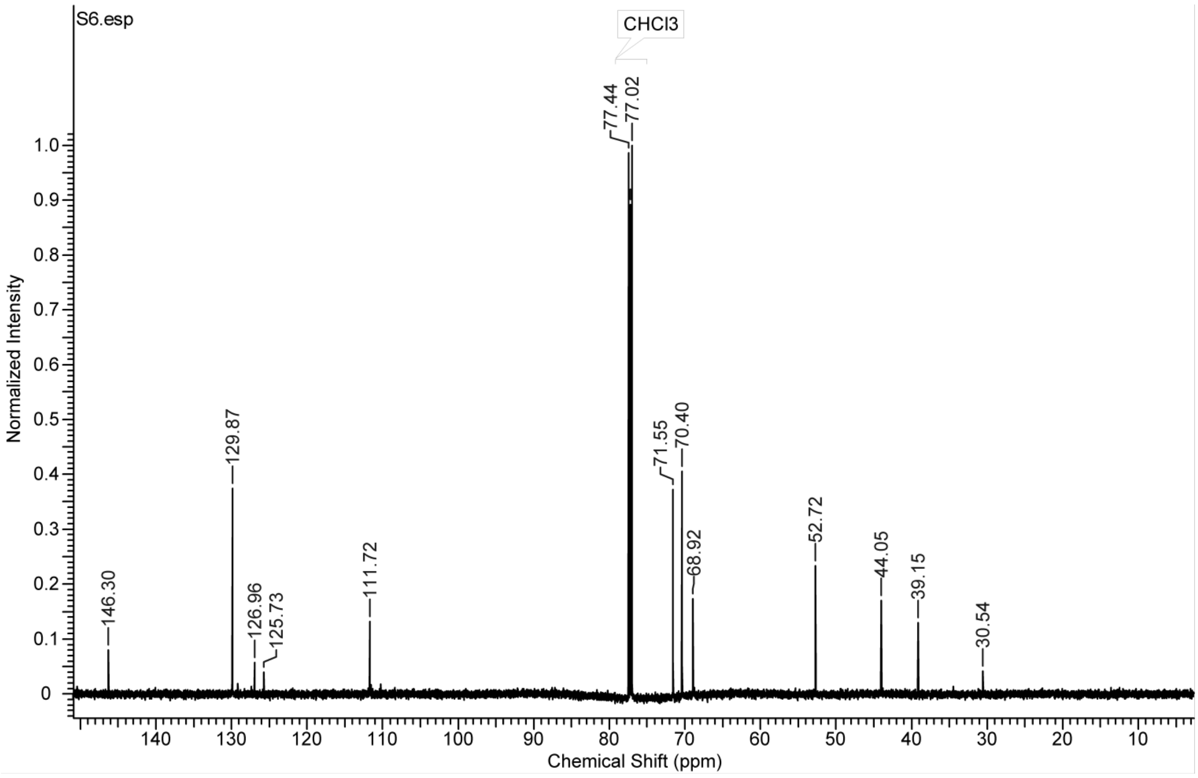

Figure S8. 600 MHz ^{1}H NMR spectrum of FI in $CDCl_3$.

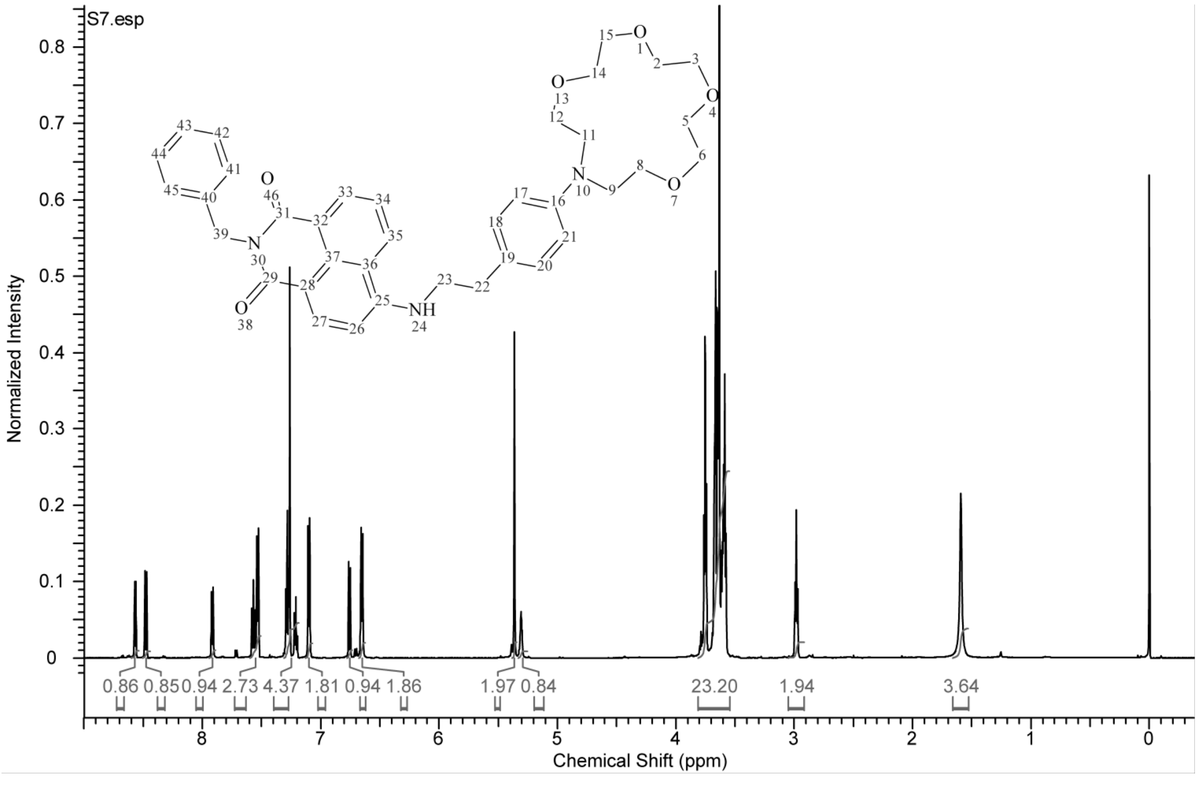

Figure S9. 151 MHz ^{13}C NMR spectrum of *N*-benzyl-[4-(aza-15-crown-5)-phenylethylamino]-1,8-naphthalimide (FI) in $CDCl_3$.

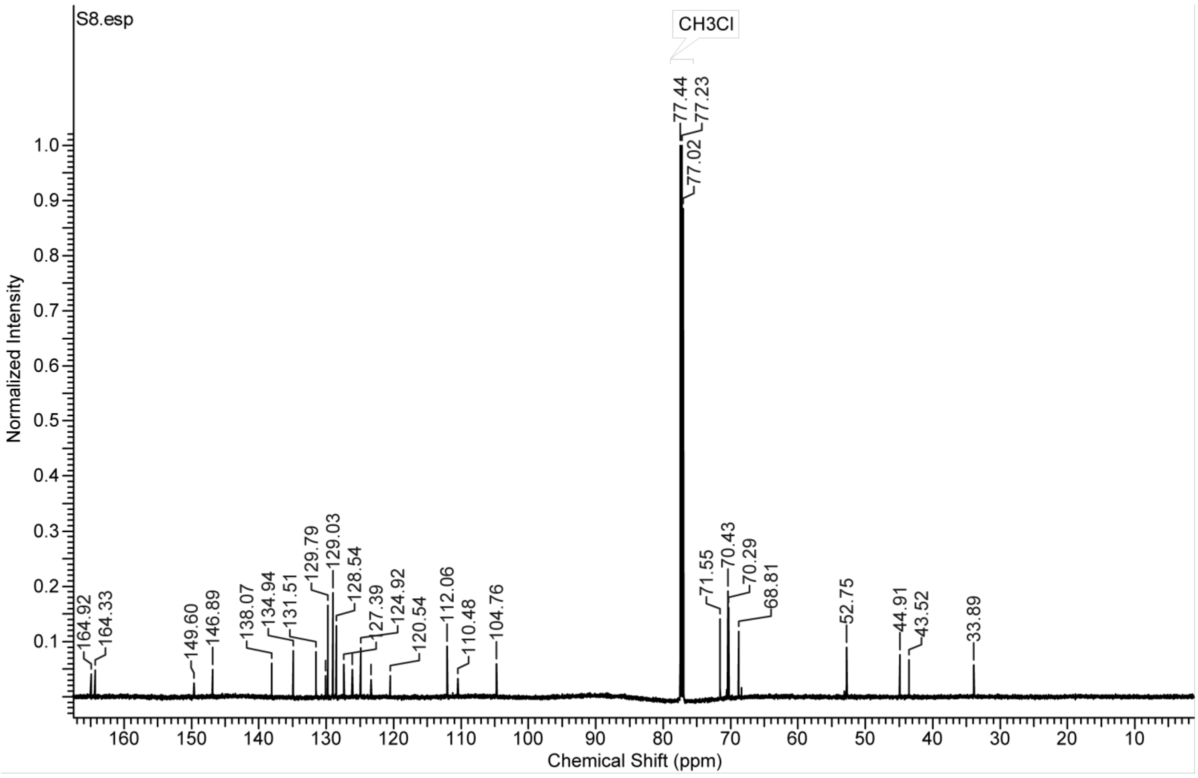

5. References

1. Leonard, J.; Procter, N.L.G. *Advanced Practical Organic Chemistry*; 2nd ed.; CRC Press: Boca Raton, FL, USA, 1994.
2. He, H.; Mortellaro, M.A.; Leiner, M.J.P.; Young, S.T.; Fraatz, R.J.; Tusa, J.K. A Fluorescent Chemosensor for Sodium Based on Photoinduced Electron Transfer. *Anal. Chem.* **2003**, *75*, 549-555.
3. Ioannidis, M.; Gentleman, A.; Ho, L.; Lincoln, S.F.; Sumby, C.J. Supramolecular Lariat Ether Binding of a Sulfonamide Aza-15-crown-5 Derivative. *Inorg. Chem. Commun.* **2010**, *13*, 593-598.
4. Optical Glass. Available online: http://www.schott.com/advanced_optics/english/our_products/materials/optical_glass.html (accessed on 4 August 2011).
5. Ebendorff-Heidepriem, H.; Monro, T.M. Extrusion of Complex Preforms for Microstructured Optical Fibers. *Opt. Express* **2007**, *15*, 15086-15092.

© 2011 by the authors; licensee MDPI, Basel, Switzerland. This article is an open access article distributed under the terms and conditions of the Creative Commons Attribution license (http://creativecommons.org/licenses/by/3.0/).
